# Expression of canonical WNT/β-CATENIN signaling components in the developing human lung

**DOI:** 10.1186/1471-213X-12-21

**Published:** 2012-07-30

**Authors:** Mingfeng Zhang, Jueping Shi, Yide Huang, Laijun Lai

**Affiliations:** 1 Fujian Key Laboratory of Developmental Biology and Neurobiology, College of Life Sciences, Fujian Normal University, Qishan Campus, Fuzhou, 350108, People's Republic of China; 2 Department of Allied Health Sciences, University of Connecticut, Storrs, CT, 06269, USA

**Keywords:** WNT/β-CATENIN signaling, Canonical, Mammalian lung development

## Abstract

**Background:**

The WNT/β-CATENIN signaling cascade is crucial for the patterning of the early lung morphogenesis in mice, but its role in the developing human lung remains to be determined. In this study, expression patterns of canonical WNT/β-CATENIN signaling components, including WNT ligands (*WNT2*, *WNT7B*), receptors ( *FZD4*, *FZD7*, *LRP5*, *LRP6*), transducers ( *DVL2*, *DVL3*, *GSK-3β*, *β-CATENIN*, *APC*, *AXIN2*), transcription factors ( *TCF4*, *LEF1*) and antagonists ( *SOSTDC1*) were examined in human embryonic lung at 7, 12, 17 and 21 weeks of gestation (W) by real-time qRT-PCR and in situ hybridization.

**Results:**

qRT-PCR analysis showed that some of these components were gradually upregulated, while some were significantly downregulated from the 7 W to the 12 W. However, most components reached a high level at 17 W, with a subsequent decrease at 21 W. In situ hybridization showed that the canonical WNT ligands and receptors were predominantly located in the peripheral epithelium, whereas the canonical WNT signal transducers and transcription factors were not only detected in the respiratory epithelium, but some were also scattered at low levels in the surrounding mesenchyme in the developing human lung. Furthermore, Western blot, qRT-PCR and histological analysis demonstrated that the β-CATENIN-dependent WNT signaling in embryonic human lung was activated in vitro by CHIR 99021 stimulation.

**Conclusions:**

This study of the expression patterns and in vitro activity of the canonical WNT/β-CATENIN pathways suggests that these components play an essential role in regulation of human lung development.

## Background

The lung arises from a small diverticulum in the anterior foregut endoderm at the laryngotracheal groove. The respiratory epithelium then invades the surrounding mesenchyme, followed by the formation of respiratory bronchioles and alveolar ducts [1,2]. Human lung morphogenesis takes place at approximately four weeks of gestation and continues into postnatal life up to early adulthood. The events of growth and development of human lung have traditionally been divided into five stages according to changes in the structure of the airway tubes and morphological modifications of epithelial cells: embryonic stage (0–7 weeks in utero), pseudoglandular stage (7–17 weeks in utero), canalicular stage (17–27 weeks in utero), saccular stage (28–36 weeks in utero) and alveolar stage (36 weeks in utero to 2 years of age) [3].

The process of lung development relies on the precise coordination of epithelial -mesenchymal interactions controlled by a number of complex signaling cascades, including bone-morphogenic proteins (BMPs), fibroblast growth factors (FGFs), sonic hedgehog (SHH) and the wingless-type MMTV integration site family (WNT) [1,4], which is known to regulate these interactions by the means of autocrine and paracrine processes [5,6]. At least three WNT signaling pathways are involved in the signal transduction process, of which the canonical WNT/β-CATENIN signaling pathway is the best characterized [7]. In the unstimulated canonical WNT/β-CATENIN pathway, β-CATENIN is bound to the scaffold proteins axin and adenomatosis polyposis coli (APC), constitutively phosphorylated by casein kinase I and glycogen synthase kinase (GSK-3β) and subsequently degraded in the cytoplasm. However, binding of the WNT ligands to two distinct membrane receptors, the frizzled (FZD) and low-density lipoprotein receptor-related proteins (LRP) 5 and 6, leads to the phosphorylation of the cytoplasmic region of LPR6 by GSK-3β and casein kinase-γ, which results in the recruitment of the cytosolic proteins known as dishevelled (DVL) 1–3 and axin. Subsequently, β-CATENIN is neither phosphorylated nor degraded, but accumulates in the cytoplasm, where it translocates into the nucleus and regulates expression of target genes such as *cyclin D1MMP7* and *c-Myc* through interactions with T cell-specific transcription factor (TCF) and lymphoid enhancer-binding factor (LEF) [7].

Many studies have used mouse genetic approaches to show that canonical WNT signaling plays an important role in the development of mammalian lung. For instance, the *WNT2* knockout mouse is associated with perinatal lethality resulting from lung hypoplasia, which is characterized by dilated endothelial vasculature, decreased cell proliferation and downregulation of genes crucial for normal lung development [8]. Mouse *WNT2* and *WNT2b* double mutants exhibit under-development of the lung including the absence of tracheal budding at E9.5 (embryonic day 9.5) and lack of certain epithelial cell markers (TTF-1 and p63) [8]. *WNT7b* knockout mice die shortly after birth due to severe lung hypoplasia with defects in branching morphogenesis and cell proliferation, as well as defects in lung epithelial differentiation. Smooth muscle α-actin expression is also abnormal in *WNT7b* mutants [9]. Similarly, inactivation of *β-CATENIN* in lung epithelium after lung budding causes aberrant epithelial branching and proximal-distal patterning [9,10]. Inactivation of *β-CATENIN* in lung mesenchyme results in decreased mesenchymal growth and defective endothelial differentiation [11]. Moreover, the deletion of *β-CATENIN* during trachea/lung morphogenesis results in shortening of the trachea and reduced lung size [12].

It has also been reported that canonical WNT/β-CATENIN signaling ligands (WNT2, WNT7B) [8,9,13], receptors (FZD4, FZD7, LRP5, LRP6) [14,15], transducers (DVL2, DVL3, GSK-3β, β-CATENIN, APC, AXIN2) [16-19], as well as transcription factors (TCF4, LEF1) [18,20] exhibit highly cell-specific expression patterns in the developing murine lung. However, tissue-specific expression of certain components involved in the canonical WNT/β-CATENIN signaling pathway during human lung development has not yet been investigated. This study demonstrated that canonical WNT signaling components are expressed in specific spatio-temporal patterns in the developing human lung by using real-time qRT-PCR analysis and in situ hybridization. Analysis of in vitro activity stimulated by CHIR 99021 further revealed that the WNT/β-CATENIN signaling cascade is crucial for early human lung patterning during morphogenesis.

## Results

### Expression of canonical WNT/β-CATENIN signaling component mRNA in the developing human lung

Human lung development can be divided into five stages with distinct structures visible at each stage. The most significant growth phase occurs in the pseudoglandular stage (7–17 weeks in utero), followed by the canalicular stage (17–27 weeks in utero). Therefore, as we previously described [21], the current study was focused on events at 7 W, 12 W, 17 W and 21 W to analyze patterns of gene expression in the developing human lung.

**Figure 1:**
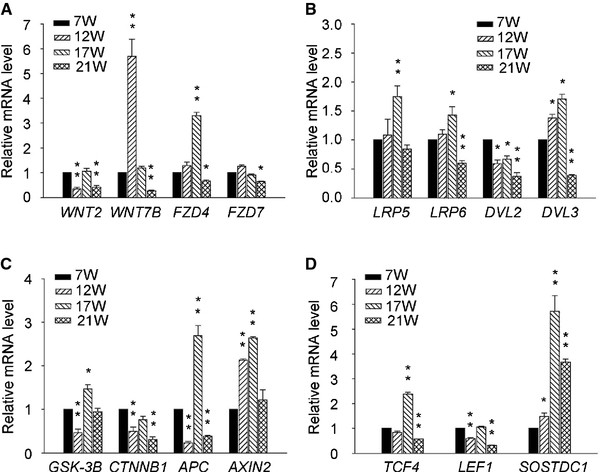
**Real-time qRT-PCR was performed to examine the mRNA expression levels (mean ± sem) of canonical WNT/β-CATENIN signaling components, including WNT ligands (*WNT2 *, *WNT7B *), WNT receptors ( *FZD4 *, *FZD7 *, *LRP5 *, *LRP6 *), WNT transducers ( *DVL2 *, *DVL3 *, *GSK-3β *, *β-CATENIN *, *APC *, *AXIN2 *), WNT transcription factors ( *TCF4 *, *LEF1 *) and WNT inhibitors or antagonists ( *SOSTDC1 *) in the developing human lung at 7 W, 12 W, 17 W and 21 W (W: Weeks of gestation) (A-D). **Data were normalized to the average mRNA level of *β*-actin at 7 W. Statistical difference is indicated by asterisks (* *P *< 0.05, ** *P *< 0.01).

Quantification of the mRNA expression of canonical WNT/β-CATENIN signaling components in human lung tissues at 7 W, 12 W, 17 W and 21 W was performed using real-time qRT-PCR. Investigation of the transcription levels of the canonical WNT ligands, *WNT2* and *WNT7B*, revealed that *WNT2* expression decreased significantly from 7 W to 12 W with a subsequent gradual increase at 17 W and a further dramatic decrease at 21 W (Figure 1A). In contrast, *WNT7B* transcripts were markedly upregulated from 7 W to 12 W, while a decreasing trend in *WNT7B* mRNA expression was observed from 12 W to 21 W (Figure 1A).

Expression of canonical WNT receptors (*FZD4*, *FZD7*) and co-receptors ( *LRP5*, *LRP6*) was also detected in human embryonic lung tissues. An obvious increase in *FZD4*, *LRP5* and *LRP6* mRNA levels was detected from 7 W to 17 W, whereas no changes in *FZD7* transcripts were observed during this period. Interestingly, mRNA levels of four canonical WNT receptor genes ( *FZD4*, *FZD7*, *LRP5*, *LRP6*) substantially decreased from the 17 W to 21 W (Figure 1A and B).

Expression of canonical WNT signal transducers (*DVL2*, *DVL3*, *GSK-3β*, *β-CATENIN*, *APC*, *AXIN2*) was also determined by qRT-PCR in the developing human lung. With exception of *DVL3* and *AXIN2*, the mRNA levels of *DVL2*, *GSK-3β*, *β-CATENIN* and *APC* were significantly downregulated from 7 W to 12 W, while *DVL3* and *AXIN2* expression markedly increased during this period (Figure 1B and C). Subsequently, the expression of canonical WNT transducers (*DVL2*, *DVL3*, *GSK-3β*, *β-CATENIN*, *APC*, *AXIN2*) increased at 17 W, but decreased at 21 W (Figure 1B and C).

Finally, the mRNA expression levels of canonical WNT signaling transcription factors (*TCF4*, *LEF1*) were examined in human lung at 7 W, 12 W, 17 W and 21 W. *TCF4* and *LEF1* presented a similar expression pattern in the developing human lung, with gradually decreasing expression levels from 7 W to 12 W followed by an obvious increase at 17 W and a further significant decrease at 21 W (Figure 1D). Analysis of expression of the WNT signaling antagonist *SOSTDC1* in embryonic human lung tissues revealed that *SOSTDC1* transcripts were upregulated from 7 W to 12 W, steadily increased to a high level at 17 W and subsequently declined at 21 W (Figure 1D).

In combination, these real-time qRT-PCR data demonstrated that most canonical WNT/β-CATENIN signaling components expressed in the developing human lung and, with the exception of *WNT7B* and *FZD7*, reached high levels at 17 W, subsequently decreasing at 21 W.

### Expression pattern of canonical WNT/β-CATENIN signaling components in the developing human lung

**Figure 2:**
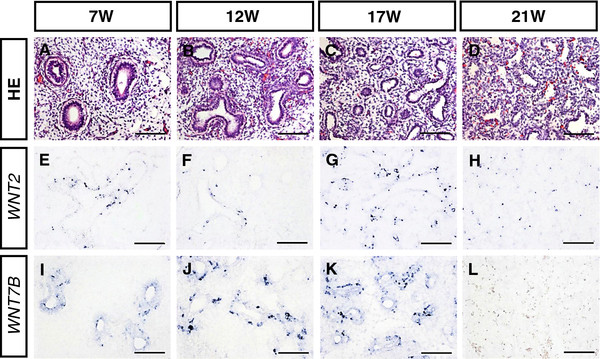
**Expression of canonical WNT/β-CATENIN signaling ligands in the developing human lung. **( **A**- **D**) Morphogenesis of the early human lung development at 7 W, 12 W, 17 W and 21 W (W: Weeks of gestation). *WNT2 *( **E**- **H**) and *WNT7B *( **I**- **L**) expression was detected in the epithelium of human lungs at 7 W, 12 W, 17 W and 21 W. Scale bars = 100 μm in **A**- **L**.

In situ hybridization was performed to confirm the expression patterns of canonical WNT/β-CATENIN signaling components during human lung development at 7 W, 12 W, 17 W and 21 W. Initial H&E staining of histological sections showed that, during the period from 7 W to 21 W (the pseudoglandular to the canalicular stage), the airway and vascular networks continuously branched and the airway epithelial cells differentiated progressively from tall columnar cells, to short columnar cells and then to cuboidal cells (Figure 2A–D). These data indicate normal lung morphogenesis and are consistent with our previous reports [21].

The mRNA expression patterns of canonical WNT ligands (*WNT2*, *WNT7B*) were then examined in the developing human lung by in situ hybridization. *WNT2* expression was obviously restricted to epithelial cells of the fetal lung at 7 W and 17 W but was dramatically downregulated at 12 W and 21 W (Figure 2E–H). *WNT7B* transcripts were clearly detected in the respiratory airways from 7 W to 17 W (Figure 2I–K) but were barely detectable at 21 W (Figure 2L).

### Expression of canonical WNT signaling receptors in the developing human lung

**Figure 3:**
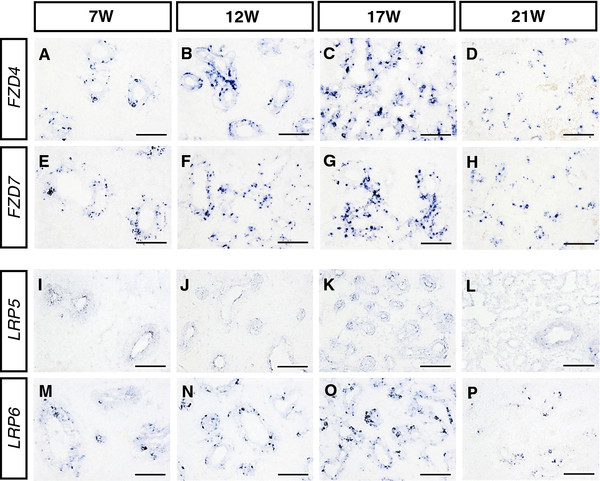
**Expression of canonical WNT/β-CATENIN signaling receptors in the developing human lung.** The expression of canonical WNT signaling receptors, including *FZD4 *( **A**- **D**), *FZD7 *( **E**- **H**), *LRP5 *( **I**- **L**) and *LRP6 *( **M**- **P**), was clearly detected in the distal part of human lung epithelium at 7 W, 12 W, 17 W and 21 W (W: Weeks of gestation). Scale bars = 100 μm in **A**- **P**.

In situ hybridization was also used to determine the expression patterns of canonical WNT/β-CATENIN signaling receptors (*FZD4*, *FZD7*) and co-receptors ( *LRP5*, *LRP6*) in the developing human lung. *FZD4*, *FZD7*, *LRP5* and *LRP6*, which exhibited very similar expression patterns, were initially restricted to the conducting airways and the peripheral epithelium from 7 W to 17 W (Figure 3A–C, E–G, I–K and M–O). Expression was subsequently maintained at a relatively low level in the peripheral epithelium at 21 W (Figure 3D, H, L and P).

### Expression of canonical WNT signaling transducers in the developing human lung

**Figure 4:**
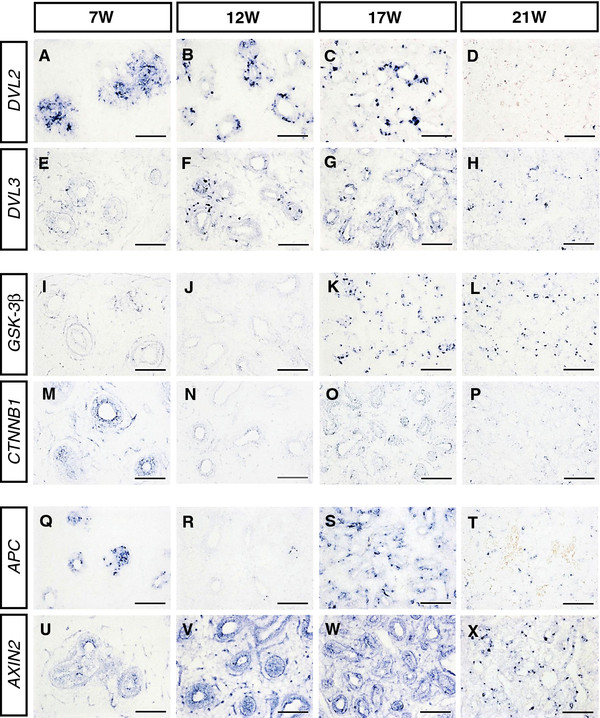
**Expression of canonical WNT/β-CATENIN transducers in the developing human lung. **Sections of the human embryos at the gestational ages of 7 W, 12 W, 17 W and 21 W (W: Weeks of gestation) showed particular expression of canonical WNT signal transducers *DVL2* ( **A**- **D**), *GSK-3β *( **I**- **L**) and *APC *( **Q**- **T**) in the distal epithelium. However, the expression of WNT signal transducers *DVL3 *( **E**- **H**), *CTNNB1 *(also *β-CATENIN*, **M**- **P**) and *AXIN2 *( **U**- **X**) was detected mainly in the distal epithelium and also weakly in the mesenchyme of human embryonic lungs at the gestational age of 7 W, 12 W, 17 and 21 W. Scale bars = 100 μm in **A**- **X**.

The mRNA expression pattern of canonical WNT/β-CATENIN signal transducers *DVL2*, *DVL3*, *GSK-3β*, *β-CATENIN*, *APC* and *AXIN2* was analyzed in fetal human lung by in situ hybridization. Expression of *DVL2*, *GSK-3β* and *APC* was distinctively confined to the peripheral epithelium from the pseudoglandular to the canalicular stage (Figure 4A–D, I–L and Q–T), with the exception of *GSK-3β* transcripts, which were detected at lower levels in both the respiratory epithelium and the pulmonary mesenchyme at 7 W (Figure 4I). In contrast, three other transducers, *DVL3*, *β-CATENIN* and *AXIN2*, were expressed strongly in the peripheral epithelium and weakly throughout the mesenchyme surrounding the terminal buds from the pseudoglandular to the canalicular stage (Figure 4E–H, M–P and U–X).

The strength of the signals attained by in situ hybridization showed that *DVL2*, *DVL3* and *AXIN2* expression remained relatively high from 7 W to 17 W (Figure 4A–C, E–G and U–W) and was obviously downregulated at 21 W (Figure 4D, H and X). Interestingly, the signals of *GSK-3β*, *β-CATENIN* and *APC* transcripts appeared strong at 7 W and 17 W (Figure 4I, K, M, O, Q and S), but were dramatically reduced and almost undetectable at 12 W and 21 W (Figure 4J, L, N, P, R and T).

### Expression of canonical WNT signaling transcription factors in the developing human lung

**Figure 5:**
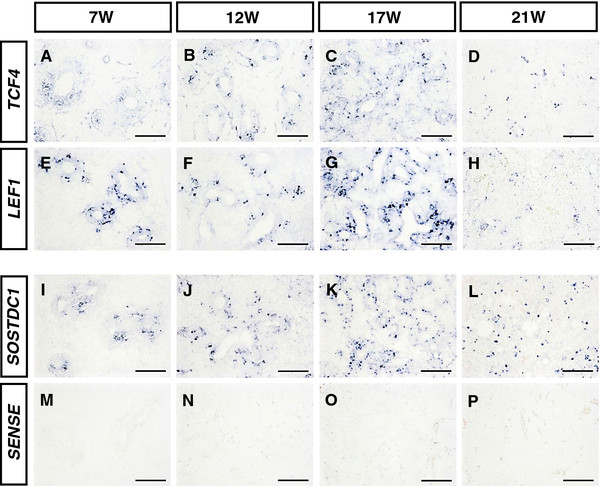
**Expression of canonical WNT/β-CATENIN signaling transcription factors in the developing human lung. **( **A**- **D**) Expression of canonical WNT signaling transcription factor *TCF4 *was detected in both the distal epithelium and the lung mesenchyme of human embryos at 7 W, 12 W, 17 W and 21 W (W: week of gestation). ( **E**- **H**) *LEF1 *staining of in situ hybridization showed a strong reactivity in the distal epithelium of the lung at 7 W, 12 W, 17 W and 21 W in human embryos. ( **I**- **L**) Sections with *SOSTDC1 *staining showed particular expression patterns in the distal lung epithelium of the embryos at 7 W, 12 W, 17 W and 21 W (W: week of gestation). ( **M**- **P**) Negative control for in situ hybridization in the developing human lung. Scale bars = 100 μm in **A**- **P**.

The distribution of canonical WNT/β-CATENIN signaling transcription factors (*TCF4*, *LEF1*) was examined during human lung development by in situ hybridization. *TCF4* was highly expressed in the peripheral epithelium and in small amounts in the surrounding mesenchymal cells during the pseudoglandular stage (Figure 5A–C), while *LEF1* was predominantly expressed in the respiratory epithelium during this period (Figure 5E–G). Subsequently, *TCF4* and *LEF1* expression was downregulated significantly in both the peripheral epithelium and the mesenchyme at the canalicular stage (Figure 5D–H). Furthermore, expression of WNT signaling inhibitor or antagonist *SOSTDC1* was also detected by in situ hybridization. Positive *SOSTDC1* staining was predominantly localized in the peripheral epithelium during the pseudoglandular stage (Figure 5I–K) and persisted into the canalicular stage (Figure 5L). Negative controls for in situ hybridization in the developing human lung were shown in Figure 5M–P.

All the in situ hybridization data obtained in this study showed that expression of canonical WNT/β-CATENIN signaling components was mainly localized in the bronchial and alveolar epithelium in embryonic human lung tissues, although some of the components were found to be expressed at low levels in the surrounding mesenchyme. Moreover, the expression patterns of canonical WNT/β-CATENIN signaling components detected by in situ hybridization were found to be in accordance with those detected by qRT-PCR.

### Activity of canonical WNT/β-CATENIN signals in the developing human lung

**Figure 6:**
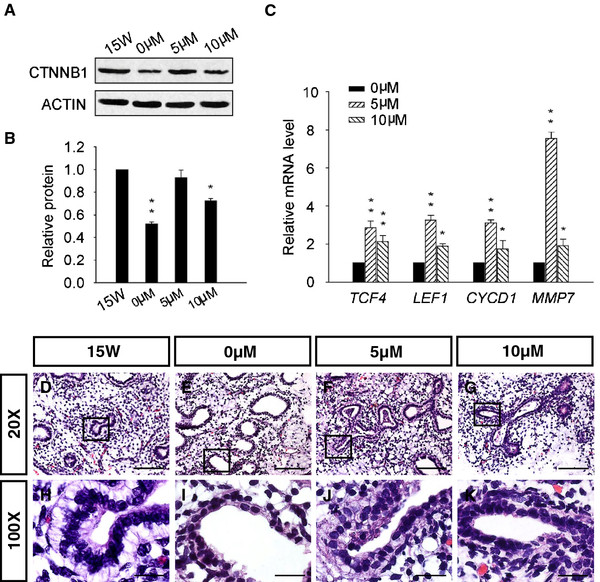
**Activity of canonical WNT/β-CATENIN signals induced by CHIR 99021 in the developing human lung. **Human lung tissue explants obtained at 15 weeks of gestation were exposed to 0, 5 and 10 μM CHIR 99021 in vitro for 72 h. ( **A**- **B**) Western blot analysis showed expression of β-CATENIN in human lung tissues. Results are presented as the mean ± sem of three independent experiments. * *P* < 0.05, ** *P *< 0.01. ( **C**) qRT-PCR analysis detected a dose-dependent increase in WNT signaling transcription factors and target genes following exposure to CHIR 99021. Results are presented as the mean ± sem of three independent experiments. * *P *< 0.05, ** *P *< 0.01. ( **D**- **K**) Histology examination showed obvious morphogenetic changes of the airway epithelial cells associated with CHIR 99021 treated lung tubes. Scale bars: **D**- **G** =100 μm; **H**- **K **= 20 μm.

To further assess canonical WNT signaling activities in the developing human lung, CHIR 99021 was used to activate WNT/β-CATENIN signaling cascades [22] through in vitro exposure of human lung explants (15 W) to 0, 5 and 10 μM CHIR 99021 for 72 h. As presented in Figure 6A and B, Western blot analysis showed β-CATENIN expression in 15 W human lung explants decreased significantly from newly isolated to in vitro cultured for 72 h (0 μM). However, β-CATENIN expression nearly increased back to newly isolated levels in human lung explants at 15 W treated with 5 μM CHIR 99021 for 72 h, but decreased again after treatment of 10 μM CHIR 99021. Meanwhile, qRT-PCR analyses showed increased expression of transcription factors (*LEF1TCF4*) and target genes ( *CYCLIN D1MMP7*) in human lung explants (15 W) exposed to 5 μM CHIR 99021 compared with tissues exposed to 0 μM CHIR 99021, the levels of which were also higher than in the 10 μM CHIR 99021 groups (Figure 6C).

We then performed histological analysis. The human lung explants (15 W) cultured in vitro for 72 h showed enlarged lung tubes and differentiation of the airway epithelial cells around the tubes from short columnar to cuboidal morphology (Figure 6D–E and H–I). However, parallel explant tissues treated with 5 μM CHIR 99021 for 72 h differentiated back to short columnar cells (Figure 6F and J), with slighter changes between cuboidal and short columnar shape observed in the groups exposed to 5 μM CHIR 99021 (Figure 6G and K). In contrast, no obvious differences were observed in the amount of lung tubes formed in the control and CHIR 99021 treated explant tissues.

## Discussion

### Crucial roles of canonical WNT/β-CATENIN signaling components in the developing human lung

This study described the mRNA expression of canonical WNT/β-CATENIN signaling components at the tissue level during human lung development at 7 W, 12 W, 17 W and 21 W. Most of the canonical WNT signaling components were detected at 7 W and increased to high levels at 17 W followed by a decrease at 21 W using quantitative real-time qRT-PCR. Expression of all essential components was demonstrated in the fetal lung and shown to be predominantly localized to the respiratory and peripheral epithelium by in situ hybridization.

Human lung development can be divided into five stages [3]. At around 4 weeks of gestation, the lung develops as an outgrowth from the ventral wall of the foregut, with trachea subsequently branching into the lobar and segmental bronchi. At approximately 7 weeks of gestation, the airways further branched and epithelial cells differentiated progressively into tall pseudostratified columnar epithelium. By 12 weeks, many small tubular structures surrounded by short columnar cells were distributed throughout the human lung sections. From 17 weeks of gestation, the short columnar epithelium was replaced progressively by cuboidal cells in a distal to proximal direction. At 21 weeks of gestation, the differentiation of pneumocytes and some extent of alveolization could be observed in human lung [3,21]. All in all, in human lung, 70 % of the total airways generated at birth are formed by 14 W [23] and all of the conducting airways and terminal bronchioles are formed by the end of 17 W [24]. Therefore, it was speculated that most of the canonical WNT signaling components were already present in the lung buds, but their functions may be predominantly involved in branching and division of conduction airway or terminal bronchioles during the pseudoglandular stage (7–17 weeks in utero). This hypothesis is also supported by previous reports that the WNT signaling system is not necessary for the establishment of the primary branching pattern of the lung but is required for appropriate branching morphogenesis [25].

Analysis of gene expression patterns indicated that all of the canonical WNT/β-CATENIN signaling components were mainly expressed in the peripheral epithelium, although the canonical WNT signal transducers and transcription factors were also slightly scattered into the surrounding mesenchyme in the developing human lung. Correct patterning of lung tissues depends on epithelial-mesenchymal cell interactions [6]. Therefore, it is possible that the canonical WNT signals are primarily transduced into the epithelial cells through WNT receptors and co-receptors and then activated by downstream signal transducers and transcription factors both in the peripheral epithelium and mesenchymal cells through epithelial-mesenchymal cell interactions. It is also possible that the fast and progressive differentiation of tubular structures and epithelial cells during human lung development results in the slighter and weaker strength of signals in the mesenchyme attained by in situ hybridization, consistent with our previous observations [21].

CHIR 99021 is one of a new class of highly selective GSK-3 inhibitors that effectively inhibit GSK-3 activity under many conditions in isolated cells and tissues [26]. In this study, CHIR99021 was used to activate canonical WNT/β-CATENIN signaling components in the developing human lung. Increased expression of β-CATENIN and WNT signal transcription factors (*TCF4LEF1*) and target genes ( *CYCD1MMP7*) was observed in human lung tissues (15 W) after exposure to CHIR 99021. Königshoff et al. also demonstrated increased mRNA levels of the WNT target gene *CYCD1* in response to WNT signal activation by WNT3A in human lung tissues in vitro [27]. Furthermore, additional activities of WNT signaling in human lung tissues (15 W) stimulated by CHIR 99021 did not accelerate the branching and division of airway conduction, but activated the airway epithelial cells retarding back from cuboidal to short columnar cells, which was contrary to the normal lung morphogenesis that the airway epithelial cells differentiated progressively from tall columnar cells, to short columnar cells and then to cuboidal cells [21].

Wnt/β-catenin signaling is one of the critical developmental pathways that are considered important for both self-renewal and differentiation of stem/progenitor cells [28]. During lung epithelial regeneration, the Wnt signaling controls the balance between stem/progenitor expansion and epithelial differentiation. Forced activation of canonical Wnt signaling leads to increased bronchioalveolar stem cell (BASCs) expression and decreased epithelial differentiation [29]. Wnt signaling has also been implicated in specifying early lung endoderm progenitors. Activation of Wnt/β-catenin signaling can reprogram posterior endoderm to a lung progenitor fate [8]. Moreover, β-catenin maintained airway epithelial progenitor cells in relatively undifferentiated state, and stabilization of β-catenin in Clara cell blocked postnatal secretory cell maturation and secretory-to-ciliated cell differentiation [28]. Therefore, in our studies, it is possible that these additional WNT signaling activities reprogram the airway epithelial cells in human embryonic lung to the early epithelial cells, which indicated the roles of WNT signaling pathway not only in appropriate lung branching morphogenesis, but also in lung cell fate decisions and differentiation. Furthermore, a WNT signaling feedback mechanism may exist in human lung tissues for expression of β-CATENIN, WNT signaling transcription factors and target genes, and differentiation of airway epithelial cells all decreased in the presence of relatively high CHIR 99021 concentrations.

### Comparison of canonical WNT/β-CATENIN signaling expression patterns in embryonic mouse and human lung

WNT signaling is known to regulate murine lung specification and development by appropriate spatial and temporal mechanisms [5]. Several WNT ligands, receptors and components of the canonical pathway are widely expressed in the developing murine lung. For instance, WNT2 is highly expressed in the developing lung mesenchyme [8], while WNT7b is predominantly localized in distal and proximal bronchial epithelial cells [9,13]. In addition, a wide range of WNT receptors including FZD4, FZD7, LRP5, and LRP6 are expressed in tissue-specific patterns during murine lung development. FZD4 and FZD7 are expressed primarily in the developing lung mesenchyme, whereas LRP5 and LRP6 are expressed in the airway epithelium of lung tissues [14,15]. In this study, expression of *WNT2WNT7BFZD4FZD7LRP5* and *LRP6* in the developing human lung was distinctly restricted to the alveolar and bronchial epithelium. This pattern of expression differs from that found in mouse lung.

In the stimulated canonical pathway, binding of the WNT ligands to the FZD receptors complex with LRP, leading to increased levels of cytosolic β-CATENIN and resulting in the translocation of β-CATENIN into the nucleus and regulation of target gene expression through interaction with the transcription factors, TCF and LEF [7]. In the mouse, three *DVL* genes ( *DVL1, 2, 3*) are widely, but not specifically, expressed in embryonic tissues [30]. DVL2 and DVL3 are expressed at relatively high and moderate levels respectively in lung tissues, although DVL1 expression has not been determined [19]. β-CATENIN is localized in the cytoplasm and the nucleus of the epithelium and adjacent mesenchyme in the developing mouse lung [18]. However, GSK-3β, APC and Axin2 exhibit moderate or weak expression levels, but are not specified in E14.5 mouse lung tissues [17]. In this study, other WNT signal transducers (*DVL3β-CATENEN* and *AXIN2*) were shown to be present not only in the peripheral epithelium, but also at low levels in the sub-adjacent mesenchyme during human lung development, whereas *DVL2* and *APC* were localized in the lung epithelium only.

Similar to the expression pattern of β-CATENIN, TCF and LEF1 were expressed in both adjacent mesenchyme and the proximal epithelium of the embryonic mouse lung [18,20]. However, in the developing human lung it was observed that *LEF1* was restricted to the alveolar and bronchial epithelium, while *TCF4* was expressed both in the epithelium and the mesenchyme. Taken together, these results suggest that, although the basic mechanism governing establishment of functional and structural diversity within the respiratory system is likely to be conserved among mammals [31], the canonical WNT signaling pathway components exhibit some different expression patterns in developing human and mouse lungs.

### The clinical significance of the canonical WNT signaling pathway in human lung development

In addition to its role in lung development and morphogenesis, the canonical WNT signaling pathway is also linked to the pathogenesis of human lung diseases, such as lung cancer, lung fibrosis, lung inflammation, pulmonary arterial hypertension (PAH) and bronchopulmonary dysplasia (BPD) [32]. The role of WNT signaling in various types of cancer is well established. For example, differential expression of several WNT components including WNT1, WNT2, WNT7A, DVL3, β-CATENIN and APC, have been reported in normal lung tissues and lung cancers, particularly in NSCLC (non-small cell lung cancer) [33-36]. Studies in animal models, as well as in human disease, have identified several components of the canonical WNT signaling pathway, including WNT2, FZD7, LRP6, β-CATENIN and GSK-3β which are overexpressed in idiopathic pulmonary fibrosis (IPF) [27]. In contrast, the role of WNT signaling in lung inflammation is largely unexplored, although the canonical pathway activator WNT-1 is implicated in lung inflammation by being linked to stimulation of pro-MMP3 transcription [37]. PAH characterized by cellular and structural changes in pulmonary arteries, has also been shown to be associated with mutations in BMPRII via activation of the WNT signaling pathway [38]. Furthermore, gene expression analysis of pulmonary arterial resistance vessels demonstrated differential regulation of canonical and non-canonical WNT genes in PAH [39]. BPD is a chronic lung disease in infants characterized by lung injury resulting from mechanical ventilation and oxygen exposure or from defects in lung development. A role for WNT signaling in BPD is suggested by activation of the pathway during hyperoxia-induced neonatal rat lung injury [40]. Dysregulation of the canonical WNT signaling pathway leads to lung disease and therefore, this investigation of the canonical WNT/β-CATENIN pathway in humans provides both further elucidation of the pathogenesis of WNT-related human lung disease and identification of potential therapeutic targets. Furthermore, given the ability of canonical Wnt signaling to regulate stem cell/progenitor expansion and regeneration in the lung, the future to use agonists of this pathway to increase lung injury repair and regeneration may be possible.

## Conclusions

This study is the first to describe the expression patterns of the canonical WNT signaling components in the developing human lung. Real-time qRT-PCR data demonstrated most of components were detected at 7 W and increased to high levels at 17 W followed by a decrease at 21 W. All the in situ hybridization data showed that expression of canonical WNT signaling components was mainly localized in the bronchial and alveolar epithelium in embryonic human lung tissues, although some of the components were expressed at low levels in the surrounding mesenchyme. The canonical WNT signaling activity was stimulated by in vitro exposure of human lung tissues into CHIR 99021. Our data of the specific spatio-temporal patterns and in vitro activity of canonical WNT signaling in the developing human lung revealed that the WNT/β-CATENIN signaling cascade is crucial for early human lung patterning during morphogenesis.

## Methods

### Tissue preparations

Human embryos at 7 to 21 weeks gestation (7 W-21 W) were obtained from the Hospital for Women and Children of Fujian Province after legal termination of pregnancy. The use of human embryos in this study was approved by the Ethical Committee of Fujian Normal University. All donors gave written informed consent for the use of their tissues for scientific purposes. Embryos were washed in phosphate buffered saline (PBS) and the lungs were dissected and fixed by overnight immersion in 4 % paraformaldehyde (PFA) in PBS at 4 °C. The specimens were then dehydrated in a graded ethanol series and embedded in paraffin. Sections (thickness, 6 μm) were prepared and subjected to hematoxylin and eosin (H&E) staining and in situ hybridization.

### RNA isolation and real-time qRT-PCR

Total RNA was extracted from 50-100 mg human embryonic tissues with TRIzol (Invitrogen, CA, USA) according to the instructions provided by the manufacturer. Template cDNAs were obtained by reverse transcription of total RNAs using oligo (dT) primer and superscript II reverse transcriptase (TAKARA, Japan). Amplification was carried out using SYBR Green qPCR Master Mix (TAKARA). Sequences of the real-time qRT-PCR primers used are listed in Table 1.

**Table 1:** Primer sequences and amplicon sizes for real-time PCR

**Gene**	**Accession**		**Sequences (5’ → 3’)**	**Length**	**Amplicon**
WNT2	NM_003391	For	CTGACCTGATGCAGACGCAAG	21 bp	139 bp
		Rev	AGGAGCCACCTGTAGCTCTCATGTA	25 bp	
WNT7B	NM_058238	For	GTCAGGGATGTTTGTCCCACTTG	23 bp	69 bp
		Rev	TCTGGTAGGTCCTTGTGCCACTC	23 bp	
FZD4	NM_012193	For	TACCTCACAAAACCCCCATCC	21 bp	132 bp
		Rev	GGCTGTATAAGCCAGCATCAT	21 bp	
FZD7	NM_003507	For	GCAAAGCAGCGCAAATCTGA	20 bp	116 bp
		Rev	AACCTCTGGCTTAACGGTGTGTG	23 bp	
LRP5	NM_002335	For	ATGGGCGCCAGAACATCAA	19 bp	117 bp
		Rev	AGATGTCGATGCTGAGGTCGTG	22 bp	
LRP6	NM_002336	For	TTGTTGCTTTATGCAAACAGACG	23 bp	167 bp
		Rev	CGTTTAATGGCTTCTTCGCTGAC	23 bp	
DVL2	NM_004422	For	TGAGCAACGATGACGCTGTG	20 bp	148 bp
		Rev	GCAGGGTCAATTGGCTGGA	19 bp	
DVL3	NM_004423	For	ACAATGCCAAGCTACCATGCTTC	23 bp	109 bp
		Rev	AGCTCCGATGGGTTATCAGCAC	22 bp	
GSK-3β	NM_002093	For	TCGAGAGCTCCAGATCATGAGAA	23 bp	124 bp
		Rev	CGGAACATAGTCCAGCACCAGA	22 bp	
CTNNB1	NM_001904	For	TCTGAGGACAAGCCACAAGATTACA	25 bp	122 bp
(β-CATENIN)		Rev	TGGGCACCAATATCAAGTCCAA	22 bp	
APC	NM_001127511	For	CATGATGCTGAGCGGCAGA	19 bp	104 bp
		Rev	GCTGTTTCATGGTCCATTCGTG	22 bp	
AXIN2	NM_004655	For	GAGTGGACTTGTGCCGACTTCA	22 bp	189 bp
		Rev	GGTGGCTGGTGCAAAGACATAG	22 bp	
TCF4	NM_001083962	For	CTGCCTTAGGGACGGACAAAG	21 bp	101 bp
		Rev	TGCCAAAGAAGTTGGTCCATTTT	23 bp	
LEF1	NM_016269	For	AATGAGAGCGAATGTCGTTGC	21 bp	137 bp
		Rev	GCTGTCTTTCTTTCCGTGCTA	21 bp	
SOSTDC1	NM_015464	For	CTTGCCCCTGCCAGTGCTCC	20 bp	211 bp
		Rev	CTCGTTGTGCTGCCGGGTGT	20 bp	
β-ACTIN	NM_001101	For	CATGTACGTTGCTATCCAGGC	21 bp	250 bp
		Rev	CTCCTTAATGTCACGCACGAT	21 bp	

Quantitative real-time qRT-PCR was performed using an ABI System Sequence Detector 7300 (Applied Biosystems, Foster City, CA, USA) under the following thermocycler conditions: stage 1, 95 °C for 30s, 1 cycle; stage 2: 95 °C for 5 s and 60 °C for 31 s, 40 cycles. *β-actin* was used as an internal control for the expression levels of target genes. Relative mRNA levels were calculated in terms of the average cycle number of PCR amplification (CT value) for the target gene where Δ = CT (target gene sample) - CT ( *β-actin* sample). Furthermore, ΔΔ = Δ (target gene value for samples at 12 W, 17 W and 21 W) - Δ (target gene value for the sample at 7 W). Finally, the formula: 2^-ΔΔ^ was used to calculate the amount of RNA relative to the control.

### In situ hybridization

In situ hybridization was performed as previously described [41]. The human cDNA vectors for *WNT2WNT7BFZD7LRP6DVL2DLV3GSK-3βAPCAXIN2* and *TCF4* digoxygenin-labelled probes were amplified from cDNA templates, which were synthesized from total RNA derived from human lung at 17 W. Sequences of the PCR primers and the enzyme sites used for insertion into the pBluescriptII KS plasmid are shown in Table 2. Other digoxygenin-labelled RNA probes for in situ hybridization of lung sections were generated from the following templates: *FZD4LRP5* and *LEF1* plasmids (kind gifts from Dr. Yiping Chen of Tulane University, USA), *CTNNB1* and *SOSTDC1* plasmids (Open Biosystems, USA). Tissue sections hybridized with sense probes were used as negative controls.

**Table 2:** Primer sequences and amplicon sizes for PCR and in situ hybridization

**Gene**	**Accession**		**Sequences (5’ → 3’)**	**Enzyme sites**	**Amplicon**
WNT2	NM_003391	For	**CCGCTCGAG**TGGGAACAGTAAAGAAAGCAGAAT	XhoI	608 bp
		Rev	**TGCTCTAGA** TTAGCTCTGGAAACCTCTCTGTCA	XbaI	
WNT7B	NM_058238	For	**CCGCTCGAG**CCCGGGGTGGCAGTAGGTAGC	XhoI	664 bp
		Rev	**TGCTCTAGA**CAGCCATCCCCCTCTCCGGTAC	XbaI	
FZD7	NM_003507	For	**CCGCTCGAG**CCTCCTGCGGTGTGCTTGTC	XhoI	519 bp
		Rev	**TGCTCTAGA**CAACCAACGGGAAACCTCAGA	XbaI	
LRP6	NM_002336	For	**CCGCTCGAG**GTGGAAGGGAATAATGGAAGC	XhoI	698 bp
		Rev	**TGCTCTAGA**CCACCAGATCAAGATGCACATTTA	XbaI	
DVL2	NM_004422	For	**CCGCTCGAG**GGGCGCTCCTGGTGTGTGAC	XhoI	434 bp
		Rev	**TGCTCTAGA**GCAGCTACATGGCCCAAATCTCC	XbaI	
DVL3	NM_004423	For	**CCGCTCGAG**GGCACGCTCACTCCCTCATTCT	XhoI	464 bp
		Rev	**TGCTCTAGA**TGCTCCAGGCCCAGGGTAAAT	XbaI	
GSK-3β	NM_002093	For	**CCGCTCGAG**GGACTCCTGCCTCATGCCCCT	XhoI	527 bp
		Rev	**TGCTCTAGA**GCTCAGCCTGCTCAACACCCC	XbaI	
APC	NM_001127511	For	**CCGCTCGAG**CGCGCTTACTGTGAAACCTGTT	XhoI	723 bp
		Rev	**TGCTCTAGA**TTGCCTGTGGTCCTCATTTGTAG	XbaI	
AXIN2	NM_004655	For	**CCGCTCGAG**GCCCGAAGCTCTTGTGAACTGTCT	XhoI	650 bp
		Rev	**TGCTCTAGA**CGCAACATGGTCAACCCTCAAGA	XbaI	
TCF4	NM_001083962	For	**CCGCTCGAG**GCTCGGCTGCCCTAGTAACAA	XhoI	678 bp
		Rev	**TGCTCTAGA**TGCACACTACTTCGGCTACACAG	XbaI	

### Pseudoglandular explant cultures and CHIR 99021 treatment

Pseudoglandular explant cultures were prepared as previously described [42]. Briefly, fetal human lungs (15 W) were isolated from surrounding structures, cut into cubes (0.5-1 mm^3^) and divided into four groups. One group was dehydrated in a graded ethanol series and embedded in paraffin. The remaining three groups were cultured on an air-liquid interface using permeable supports (Transwell, 0.4-μm pore size; Corning, USA) in DMEM media in the absence or presence of CHIR 99021 (5 and 10 μM). Explants were cultured at 37 °C in 95 % air/5 % CO_2_ for up to 72 h prior to collection for analyses. A stock solution of CHIR 99021 (S1263, Selleck Chemicals, USA) was prepared at 1 mM and stored at 4 °C.

### Western blotting

Protein extracts of human lung explants (15 W) before or after CHIR 99021 treatment for 72 h were prepared according to a previously described nuclear protein extraction protocol [43]. Extracts were subjected to Western blot analysis using mouse anti-human β-CATENIN (1:1000, Millipore, USA) and mouse anti-human β-actin (1:500, Zhongshan Goldenbridge Biotechnology Co., Ltd., China) primary antibodies. Blots incubated in the absence of the primary antibodies were used as negative controls. The Polink-2 plus Polymer HRP Detection System for mouse primary antibodies (Zhongshan Goldenbridge Biotechnology Co., Ltd.) was used as the secondary antibody. Western Blotting Chemiluminscence Luminol Reagent (Santa Cruz, USA) was added to detect immunopositive protein bands. The data was obtained from triplicates of each independent experiment. Values were normalized to corresponding β-ACTIN levels and then expressed as a percentage of the control value.

### Statistical analysis

Experimental data were presented as mean ± standard error of the mean (sem) from at least three independent experiment. The data were analyzed with ANOVA accompanied by Student’s *t*-test to detect significant differences of two independent groups by using the SigmaPlot 10.0 Software. Differences with P < 0.05 or P < 0.01 were considered to be statistically significant.

## Competing interests

The authors declare that they have no competing interests.

## Authors’ contributions

The authors declare the following contributions: conceived the study and design of experiments: MFZ. Collected tissues: MFZ and JPS. Performed the experiments and analysis: MFZ, JPS and YDH. Manuscript preparation and discussion: MFZ and LJL. All authors read and approved the final manuscript.
